# Using AI to Improve Individual and Population Health

**DOI:** 10.3389/ijph.2026.1609114

**Published:** 2026-02-06

**Authors:** William B. Weeks, James N. Weinstein, Juan M. Lavista Ferres

**Affiliations:** 1 AI for Good Research Lab, Microsoft, Redmond, WA, United States; 2 Microsoft Research, Microsoft, Redmond, WA, United States

**Keywords:** public health, population health, health care delivery, artificial intelligence (AI), health outcomes

## Population Health Management and Chronic Disease Prevalence in the United States

At the intersection of public health and healthcare delivery, population health management analyzes population level data to identify opportunities to improve the quality, efficiency, and equity of care being provided. Population health management incorporates: defining a population of individuals; recognizing the circumstances in which that population is born, grows, lives, works, and ages [1]; understanding the healthcare needs of that population; and offering interventions that are targeted to individuals within that population to optimize health.

Ninety percent of United States (US) healthcare expenditures are on people with chronic health conditions [2]. In 2018, 27.2% of the adult US population had least two of ten common major chronic conditions (arthritis, cancer, chronic obstructive pulmonary disease, current asthma, diabetes, hepatitis, hypertension, stroke, or weak or failing kidneys); 24.6% had one condition; and 48.2% had none; the proportion of those with at least one chronic condition is increasing [3].

These three populations have distinct population health management goals: to keep those without any chronic diseases (the healthy population) disease-free; to manage those with one chronic medical disease (the moderately illness burdened population) so that either they enter the healthy population group, avoid additional chronic medical diseases, or do not develop sequelae of their chronic disease; and to maximize patient functionality, independence, and disease control among those with multiple chronic diseases (the substantially illness burdened population).

## How Artificial Intelligence can Improve Healthcare Delivery

A healthcare delivery system has a service population that is generally defined by geography. The healthy population will interact rarely with healthcare systems while those with increasing illness burdens will interact with them more frequently. When a patient encounters a healthcare system, evaluation, diagnostic, management, consultative, and advisory services are provided. Patients then return to the service population–perhaps moving into a different illness burden category. Supporting and orchestrating that care process are administrators and healthcare providers who operate within a political environment that policymakers can influence through interventions designed to address social determinants of health (SDOH) that adversely impact health outcomes.

The goals of healthcare delivery are to improve the patient experience, improve the provider experience, improve equitable care quality and outcomes, and reduce *per capita* healthcare costs [4]. To achieve that quadruple aim in populations with moderate and substantial illness burdens, population health management goals can use artificial intelligence (AI) to: reduce over-diagnosis and overtreatment [5]; identify fraud, waste, and abuse [6]; and identify social determinants of health (SDOH) that contribute to inferior measures of population health [7].

The Table 1 articulates how AI tools–including analysis of peripheral devices, large language models (LLMs), AI-driven predictive algorithms, chatbots, and agents–can be used to achieve population and individual health goals, for distinct populations. AI tools can support patients by monitoring health, promoting healthy decision making, and informing them about treatment options. Further, they can assist providers and administrators through clinical and operational decision support, including anticipating population healthcare needs, developing guidelines to support optimal care pathways, and modeling workforce needs.

**TABLE 1:** Strategic deployment of artificial intelligence (AI) to improve individual and population health management and stakeholder actions facilitated by that deployment. LLMs mean large language models; SDOH means social determinants of health.

Population	Individual and population health goals	Key AI tools to be deployed to help achieve those goals	Stakeholder actions facilitated by AI tool deployment
Healthy (no chronic diseases)	- Maintain health - Prevent chronic disease	- Analysis of peripherals and feedback - LLMs for preventive advice	- Providers: promote prevention - Administrators: Anticipate population health needs - Policymakers: Identify, address, and evaluate SDOH interventions that might promote chronic disease development
Moderately Ill (1 chronic disease)	- Cure/manage disease - Prevent disease progression	- AI-driven predictive algorithms - Chatbots for disease management - LLMs for preventive advice and to articulate treatment options	- Providers: Efficient care management - Administrators: Develop guidelines - Policymakers: Create incentives to steer patients to the most efficient and effective providers
Substantially Ill (2+ chronic diseases)	- Maximize functioning - Coordinate care - Maintain independence	- LLMs for care management - Agents to coordinate care - Decision support for value-based informed choice	- Providers: Coordinate complex care - Administrators: Modeling workforce needs - Policymakers: Prevent fraud, waste, and abuse, including overdiagnosis and overtreatment

Finally, AI can help policymakers identify, address, and evaluate policy interventions designed to promote positive health outcomes for populations, for example, by identifying and addressing SODH that preclude optimal individual and population health. And, while there is evidence that addressing SDOH disparities is associated with improving population health [8], policymakers can use AI analytics to evaluate the timing, magnitude, and beneficiaries of those efforts in order to replicate them. Such analyses could effectively communicate the value of the investments that have accrued to targeted populations and can identify potential funding partners who benefit from local investments designed to improve SDOH.

### Limitations

To be sure, AI is not a panacea. The European Commission has identified challenges to the use of AI for the purposes of improving individual and population health [9]. Concerns include objectively determining whether AI algorithms are: usable, effective, fair, safe and reliable, transparent, and ensure security and privacy. While these concerns are valid, we are confident that–with regulation, oversight, guardrails, and continuous monitoring–AI can effectively, usefully, reliably and safely be used, not only to improve care outcomes and quality, but also, as suggested above, to improve care access, the patient experience, and the provider experience, all while reducing *per capita* healthcare costs by avoiding unnecessary care, fraud, waste, and abuse. The current healthcare system is sub-obtimal and includes the same concerns raised by the European Commission.

It will be important to evaluate the use of AI for improving individual and population health against the current state of healthcare delivery and population health management in the United States and not against an unattainable, ideal state. We recommend piloting (on target populations), evaluation of pilot results against anticipated performance, and ongoing monitoring of the effectiveness, safety, reliability, and security of implemented AI algorithms.

### Conclusion

AI is rapidly transforming multiple sectors, including healthcare, in ways that are likely to have lasting impact. While the term “artificial intelligence” was coined by John McCarthy in 1955 [10] and AI has been integrated into navigation applications, smart phones and watches, and recommender systems, Generative Pre-trained Transformers, which became widely availble to the public with the release of ChatGPT in November 2022, are the most rapidly uptaken technologies in the history of the world. AI is transforming labor markets; it will do the same in healthcare.

A comprehensive, strategic deployment of particular AI tools that target particular patient populations, support administrators and providers, and inform policymakers can not only reduce waste but also efficiently and effectively support a learning cycle, wherein individual and population health continuously improve.
